# Prevalence of symptomatic axial osteoarthritis phenotypes in Spain and associated socio-demographic, anthropometric, and lifestyle variables

**DOI:** 10.1007/s00296-021-05038-4

**Published:** 2021-11-10

**Authors:** Maite Silva-Díaz, Francisco J. Blanco, Víctor Quevedo Vila, Daniel Seoane-Mato, Fernando Pérez-Ruiz, Antonio Juan-Mas, José M. Pego-Reigosa, Javier Narváez, Neus Quilis, Raúl Cortés, Antonio Romero Pérez, Dolores Fábregas Canales, Teresa Font Gayá, Carolina Bordoy Ferrer, Francisco Javier Prado-Galbarro, Carlos Sánchez-Piedra, Federico Díaz-González, Sagrario Bustabad-Reyes, on behalf of the Working Group Proyecto EPISER2016

**Affiliations:** 1 Unidad de Investigación Clínica, Grupo de Investigación de Reumatología (GIR), Instituto de Investigación Biomédica de A Coruña (INIBIC), Complexo Hospitalario Universitario de A Coruña (CHUAC), Sergas, Universidade da Coruña, A Coruña, Spain; 2 Universidade da Coruña (UDC), Grupo de Investigación de Reumatología y Salud (GIR-S). Departamento de Fisioterapia, Medicina y Ciencias Biomédicas, Facultad de Fisioterapia,, Campus de Oza, A Coruña, España; 3 Rheumatology Unit, Hospital Comarcal Monforte de Lemos, Monforte de Lemos, Lugo, España; 4 Research Unit, Spanish Society of Rheumatology, Madrid, España; 5 Rheumatology Department, Hospital Universitario Cruces, Baracaldo, Vizcaya, España; 6 Rheumatology Department, Hospital Son LLàtzer, Palma de Mallorca, Baleares España; 7 Rheumatology Department, Complejo Hospitalario Universitario de Vigo, Grupo IRIDIS, Instituto de Investigación Sanitaria Galicia Sur (IISGS), Vigo, Pontevedra, España; 8 Rheumatology Department, Hospital Universitario de Bellvitge, L’Hospitalet de Llobregat, Barcelona, España; 9 Rheumatology Department, Hospital General Universitario de Elda, Elda, Alicante, España; 10 Rheumatology Department, Hospital General de Ontinyent, Ontinyent, Valencia, España; 11 Rheumatology Department, Complejo Hospitalario de Jaén, Jaén, España; 12 Rheumatology Department, Hospital de Barbastro, Barbastro, Huesca, España; 13 Rheumatology Department, Hospital Comarcal de Inca, Inca, Baleares, España; 14 Orphan Drug Laboratory, Biologic System Department, Metropolitan Autonomous University, Mexico City, Mexico; 15 Department of Internal Medicine, Dermatology and Psyquiatry, Universidad de La Laguna, La Laguna, Santa Cruz de Tenerife, España; 16 Rheumatology Department, Hospital Universitario de Canarias, La Laguna, Santa Cruz de Tenerife, España

**Keywords:** Osteoarthritis, Spine, Phenotypes, Prevalence

## Abstract

**Objective:**

Axial osteoarthritis (OA) is a common cause of back and neck pain, however, few studies have examined its prevalence. The aim was to estimate the prevalence and the characteristics of symptomatic axial OA in Spain.

**Methods:**

EPISER2016 is a cross-sectional multicenter population-based study of people aged 40 years or older. Subjects were randomly selected using multistage stratified cluster sampling. Participants were contacted by telephone to complete rheumatic disease screening questionnaires. Two phenotypes were analyzed, patients with *Non-exclusive axial OA (NEA-OA)* and *Exclusive axial OA (EA-OA)*. To calculate the prevalence and its 95% confidence interval (CI), the sample design was considered and weighting was calculated according to age, sex and geographic origin.

**Results:**

Prevalence of NEA-OA by clinical or clinical-radiographic criteria was 19.17% (95% CI: 17.82–20.59). The frequency of NEA-OA increased with age (being 3.6 times more likely in patients aged 80 s or more than in those between 40 and 49 years) and body mass index. It was significantly more frequent in women, as well as in the center of Spain. It was less frequent in those with a higher level of education. Lumbar OA was more frequent than cervical OA. This difference grew with increasing age and was not associated with gender. It was also greater in overweight and obese subjects.

**Conclusions:**

This is the first study on the prevalence of axial OA phenotypes in Europe describing the associated socio-demographic, anthropometric, and lifestyle variables.

## Introduction

Osteoarthritis (OA) is a heterogeneous group of diseases with similar clinical manifestations and sharing common pathological and radiological changes. Previously OA was described as a single disease, but this is not entirely correct. OARSI has recently defined OA as a disorder that affects movable joints, one characterized by cell stress and degradation of the extracellular matrix of cartilage that begins with the presence of micro and macro lesions that activate mal-adaptive repair responses, including pro-inflammatory pathways of innate immunity. The disease initially manifests as a molecular alteration (abnormal joint tissue metabolism), which is followed by anatomical and/or physiological alterations (characterized by cartilage degradation, bone remodeling, osteophyte formation, joint inflammation and loss of normal function), which can culminate in the onset of the illness [1]. OA has been also described as joint dysfunction [2]. The joint would be like any other organ of the human body, such as the heart, with its specialized tissues that form a structure with specific functions.

Axial OA is a clinical and pathological dysfunction that involves the functional failure of the synovial facet joints. This failure process involves the whole joint, including the subchondral bone, cartilage, ligaments, capsule, synovium, and periarticular paraspinal muscles and soft tissues [3]. Kirkaldy–Willis described the spinal degenerative cascade that affects the three joint complexes comprised of the intervertebral disk (front) and the lumbar zygapophyseal (facet) joints (posterior) [4]. While most patients experience an initial alteration in the anterior spinal structures, some individuals (10–20%) exhibit a pattern of isolated posterior degeneration without substantial loss of disk height. Increased age, body mass index and female sex may be related to posterior degeneration in these individuals [5].

About half of adults suffer from neck pain and two-thirds from lower back pain at some point in their lifetime [6, 7]. Axial OA is a common cause of back and neck pain, which in turn have an enormous global impact on the health-care systems and economies of developed countries [8, 9]. Lower back and neck pain were the leading global cause of disability in 2015 in most countries [10] and they remain important causes of absenteeism and premature retirement [11].

There are few studies on the prevalence of axial OA, most of them from Eastern Asia (Korea, China and Japan). There are more publications about lumbar OA [12–17] than cervical OA [18], and some studies address both [19–21], although separately. In 2000 the Spanish Society of Rheumatology (SER) promoted the EPISER2000 study, which attempted to determine the prevalence of rheumatic diseases in people older than 20 years, although axial OA was not included. The prevalence in Spain of knee OA was estimated as 10.2% and that of hand OA 6.2% [22, 23]. The changes in socio-demographic characteristics and lifestyle habits that have occurred in recent years in Spain justified updating the epidemiological data, leading SER to promote the EPISER2016 study [24]. Our group has recently published partial results of this project [25], wherein the prevalence of symptomatic hand OA was 7.73%, knee OA was 13.83% and hip OA was 5.13%. This new project also encompassed axial OA. Based on these findings, the main objective of this work was to describe the characteristics and the prevalence of symptomatic axial OA (cervical and lumbar) in Spain. To compare we established three groups of patients: (a) Non-exclusive axial OA (NEA-OA phenotype): patients with Axial OA (cervical and/or lumbar) with or without a simultaneous peripheral OA; (b) Exclusive axial OA (EA-OA) phenotype: patients with Axial OA (cervical and/or lumbar) without a peripheral OA and c) Control: subjects aged 40 or over without axial OA used to compared with NEA-OA (these subjects can have peripheral OA) and EA-OA phenotypes (these subjects have not peripheral).

## Patients and methods

The methods and characteristics of the sample from the EPISER2016 study have been previously described [24, 26]. In summary, it is a population-based multicenter cross-sectional study to estimate the prevalence of 13 rheumatic diseases (rheumatoid arthritis, SLE, symptomatic OA of the hand, knee, hip, cervical, and lumbar spine, fibromyalgia, ankylosing spondylitis, psoriatic arthritis, Sjögren’s syndrome, gout, and symptomatic osteoporotic fracture) in the adult population (≥ 20 years old) in Spain. Assuming a Poisson distribution, a sample comprising 4,000 individuals would enable a 95% confidence interval (CI) of 0.30–0.77 for a prevalence of 0.5% (expected for rheumatoid arthritis) and of 0.14–0.54 for a prevalence of 0.3% (expected for psoriatic arthritis). Assuming that missing values would reach 20%, it was deemed necessary to include around 5000 individuals.

A multistage stratified cluster random sampling was carried out based on rural/urban municipalities, sex and age in accordance with the population distribution in Spain. Resident subjects in 78 municipalities randomly selected from the 17 Spanish autonomous communities belonging to 21 reference area hospitals participated [26]. From November 2016 to October 2017, the participants in each municipality were contacted using random digit dialing and a computer-assisted telephone interviewing system (CATI) to conduct screening questionnaires. An external sociological research company with experience in the field of health care and with call center service (Ipsos España) implemented both the random selection of telephone numbers in each municipality and the initial screening interviews. In the case of non-answered phone calls, a minimum of six attempts were made during different time frames. If after these attempts there was no answer or the subject refused to participate, another phone number within the same municipality was randomly selected [26].

**Fig. 1:**
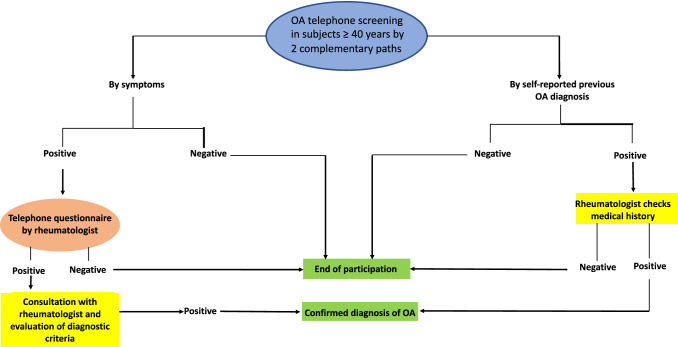
Axial OA screening algorithm

Screening was based on two complementary paths for all of the participants (Fig. 1). If a participant reported having been diagnosed, his/her consent was requested so that the investigating rheumatologists from that municipality's reference hospital could confirm the diagnosis was in his/her clinical history. Participants who met the criteria of an initial screening based on their symptoms were also identified. Participants not previously diagnosed, but who had a positive result in that symptom-based screening, received a second telephone call from the investigating rheumatologist to evaluate the suspicion by means of a second questionnaire.

Those participants for whom such suspicion lingered after the second telephone call were given an appointment at their reference hospital to complete the diagnostic confirmation process (physical examination and additional tests). Those subjects who had completed the call center interview with a positive result for axial OA screening, but whose records remained inaccessible to the rheumatologist or who could not be contacted to confirm or rule out the diagnosis were considered missing.

The study of the prevalence of OA was limited to subjects ≥ 40 years old.

A screening for symptoms of cervical or lumbar OA was considered positive if the individual had cervical or lumbar pain not due to trauma or overstrain of at least 3 months' duration (although the pain may have fluctuated in intensity) and that was aggravated by neck movements, carrying weight or making efforts. If in the second phone call the subject confirmed the characteristics of the pain previously described in the first phone call, he/she was asked about previous radiographic test to study that pain and the results of it. Suspicion remained if the individual reported an abnormal result or no previous radiological test.

These pathologies did not involve criteria approved by a specific society or scientific group. Therefore, the following criteria were specifically defined for this study to diagnose cervical and lumbar OA: (1) Cervical or lumbar mechanical pain of more than 3 months' evolution; (2) Stiffness of less than 30 min or the absence of stiffness; (3) Vertebral osteophytes or decreased intervertebral space with sclerosis of the vertebral endplates; (4) Sclerosis of the interapophyseal joints. The diagnosis was confirmed if two of the clinical criteria (1 and 2) and at least one of the radiological criteria (3 and 4) were met.

**Fig. 2:**
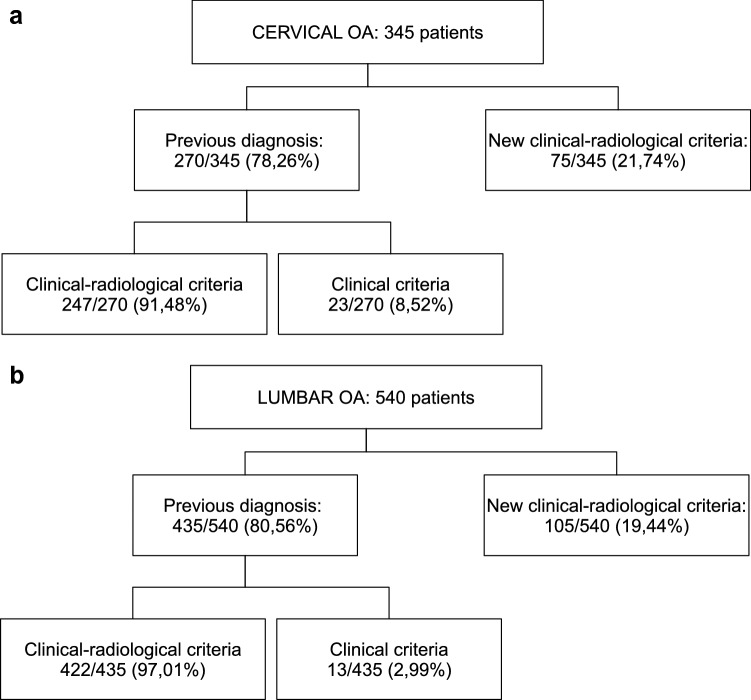
Number of cervical and lumbar OA cases based on diagnostic criteria

These criteria were used to confirm those cases not diagnosed before the study. In the case of previously diagnosed patients, no attempt was made to actively verify that they fulfilled the criteria according to their clinical history; clearly identified diagnoses were accepted irrespective of the criteria applied (clinical or clinical-radiographical) (Fig. 2).

Variables collected in the first phone questionnaire consisted of demographic characteristics (age by decades, sex, geographic area of Spain—North, Mediterranean and Canary Islands, and Center—, type of municipality—urban if at least one town exceeded 10,000 inhabitants—and educational level—basic, medium or higher), body mass index (BMI) (normal weight, low weight, overweight, obese), smoking, and alcohol intake.

Oral informed consent was required from all participants during the first telephone call and their approval was recorded on audio. Written informed consent was also requested from all subjects who came to the participating centers for physical examinations and additional tests. Approval was obtained from the Research Ethics Committee (REC) of Hospital Universitario de Canarias (approval number: Acta 12/2016), which acted as the reference REC, and from the RECs of those participating centers that required to approve the study locally. The study was performed in compliance with the principles of the Declaration of Helsinki.

### Statistical analysis

Three groups of patients were used in the statistical analysis according these definitions: (a)* Non-exclusive axial OA (NEA-OA phenotype)*: Patients with Axial OA (cervical and/or lumbar) with or without a simultaneous peripheral OA. (b) *Exclusive axial OA (EA-OA) phenotype*: Patients with Axial OA (cervical and/or lumbar) without a peripheral OA. (c) *Control:* Subjects aged 40 or over without axial OA used to compared with NEA-OA (these subjects can have peripheral OA) and EA-OA phenotypes (these subjects have not peripheral).

Prevalence and its 95% CI were calculated in accordance with the design of the sample. The weights were calculated depending on the selection probability in each of the stages of the sampling, using as a reference the distribution of the population in Spain in 2016 according to Continuous Register Statistics from the Spanish National Statistical Institute (www.ine.es). This weighting was calculated considering age (grouped by decades), sex, and geographic origin (3 areas were defined: North [Galicia + Asturias + Cantabria + País Vasco + Navarra + La Rioja], Mediterranean and Canary Islands [Cataluña + Comunidad Valenciana + Balearic Islands + Murcia + Andalucía + Canary Islands], and Center [Comunidad de Madrid + Castilla y León + Aragón + Castilla-La Mancha + Extremadura]). Based on these characteristics, each individual in the sample represented a certain number of individuals in the population.

Finally, any associations between axial OA and socio-demographic, anthropometric and lifestyle variables included in the first telephone questionnaire were analyzed. First, a bivariate analysis was carried out to determine any associations between the disease and each of the variables. Subsequently, binary logistic regression models were constructed using those variables with a *p* value of < 0.2 in the bivariate analysis (age and sex were included in the model, regardless of the *p* value in the bivariate analysis). Statistical significance was defined as *p* < 0.05.

The analyses were performed using IBM SPSS Statistics v22.

## Results

The EPISER2016 study sample for analyzing OA consisted of 3,336 subjects ≥ 40 years in age, among whom 48 were missing and 978 (29.32%) had peripheral and/or axial OA. The prevalence of OA in Spain, in one or more of the studied locations (hand, knee, hip, cervical and/or lumbar) was 29.35% (95% CI: 27.77–30.97) [25]. The mean age of the OA cases was 64.72 years; 730 were women, 62.6% had undergone basic studies, 70.1% were overweight or obese, and 83.9% were ex-smokers or non-smokers (Table 1).

**Table 1:** Descriptive analysis of the subjects with OA

	Total OA	NEA-OA	EA-OA	Exclusive cervical OA	Exclusive lumbar OA
Age range (years)					
40–49	125 (12.8)	88 (13.6)	69 (21.6)	25 (21.2)	46 (14.8)
50–59	242 (24.7)	165 (25.4)	110 (34.5)	41 (34.7)	82 (26.5)
60–69	257 (26.3)	183 (28.2)	73 (22.9)	31 (26.3)	73 (23.5)
70–79	201 (20.6)	125 (19.3)	40 (12.5)	12 (10.2)	63 (20.3)
≥ 80	153 (15.6)	88 (13.6)	27 (8.5)	9 (7.6)	46 (14.8)
Sex					
Men	248 (25.4)	144 (22.2)	83 (26.0)	31 (26.3)	77 (24.8)
Women	730 (74.6)	505 (77.8)	236 (74.0)	87 (73.7)	233 (75.2)
Region of Spain					
North	259 (26.5)	171 (26.3)	85 (26.6)	27 (22.9)	96 (31)
Mediterranean and Canary Islands	378 (38.7)	241 (37.1)	104 (32.6)	54 (45.8)	95 (30.6)
Center	341 (34.9)	237 (36.5)	130 (40.8)	37 (31.4)	119 (38.4)
Type of municipality					
Rural	212 (21.7)	134 (20.6)	65 (20.4)	23 (19.5)	63 (20.3)
Urban	766 (78.3)	515 (79.4)	254 (79.6)	95 (80.5)	247 (79.7)
Educational level					
Basic	611 (62.6)	417 (64.3)	175 (54.9)	67 (56.8)	188 (60.6)
Medium	207 (21.2)	134 (20.6)	81 (25.4)	23 (19.5)	72 (23.2)
Higher	158 (16.2)	98 (15.1)	63 (19.7)	28 (23.7)	50 (16.1)
Body mass index					
Normal weight	266 (29.4)	187 (31)	115 (37.3)	48 (42.9)	87 (30.2)
Low weight	4 (0.4)	2 (0.3)	2 (0.6)	2 (1.8)	0
Overweight	405 (44.8)	259 (43)	137 (44.5)	44 (39.3)	126 (43.8)
Obese	229 (25.3)	155 (25.7)	54 (17.5)	18 (16.1)	75 (26)
Smoking					
Non-smoker	561 (57.4)	375 (57.8)	164 (51.4)	58 (49.2)	177 (57.1)
Former smoker	259 (26.5)	161 (24.8)	82 (25.7)	29 (24.6)	82 (26.5)
Smoker	143 (14.6)	106 (16.3)	69 (21.6)	31 (26.3)	45 (14.5)
Occasional smoker	15 (1.5)	7 (1.1)	4 (1.3)	0	6 (1.9)
Alcohol (daily intake)					
0 SDU	797 (81.5)	542 (83.5)	270 (84.6)	100 (84.7)	260 (83.9)
1 SDU	120 (12.3)	73 (11.2)	37 (11.6)	12 (10.2)	33 (10.6)
2–3 SDUs	50 (5.1)	26 (4)	8 (2.5)	4 (3.4)	13 (4.2)
≥ 4 SDUs	11 (1.1)	8 (1.2)	4 (1.3)	2 (1.7)	4 (1.3)

*Total OA* hand, knee, hip, cervical and/or lumbar OA, *NEA-OA* cervical and/or lumbar OA with or without peripheral OA, *EA-OA* cervical and/or lumbar OA without peripheral OA, *SDU* standard drink unit

### Axial OA with or without a simultaneous peripheral OA phenotype (NEA-OA phenotype)

The number of cases with axial OA based on clinical or clinical-radiographic criteria in EPISER2016 was 664, which represents a prevalence of 19.17% (95% CI: 17.82–20.59); by sex, this equated to 25.31% (23.45–27.26) in women and 12.43% (10.56–14.57) in men.

There were 649 cases of axial OA that met clinical-radiographic criteria. Of these, some of which involved peripheral OA, 77.8% were women and 79.4% lived in an urban environment (Table 1). Most subjects had a basic educational level (64.3%), were overweight or obese (68.7%) and did not refer toxic habits (57.8% were non-smokers and 83.5% did not drink alcohol on a daily basis).

In the univariate analysis of cases with NEA-OA phenotype versus subjects aged 40 years or older without axial OA (although in both groups a number of patients had peripheral OA), the frequency of NEA-OA phenotype increased with age. In addition, it was more frequent in women, in inhabitants living in the center of Spain, as well as in people with a basic educational level. NEA-OA phenotype was more common in those who were obese, non-smokers, and nondrinkers (Table 2).

**Table 2:** Univariate analysis of NEA-OA vs subjects without axial OA ≥40 years old

	NEA-OA	Subjects without axial OA	*p* value*
Age range (years)			< 0.001
40–49	88 (8.5)	950 (91.5)	
50–59	165 (20.1)	657 (79.9)	
60–69	183 (27.2)	491 (72.8)	
70–79	125 (27.5)	330 (72.5)	
≥ 80	88 (30.1)	204 (69.9)	
Sex			< 0.001
Men	144 (12.1)	1046 (87.9)	
Women	505 (24.2)	1586 (75.8)	
Region of Spain			0.001
North	171 (18.4)	758 (81.6)	
Mediterranean and Canary Islands	241 (17.7)	1117 (82.3)	
Center	237 (23.8)	757 (76.2)	
Type of municipality			0.049
Rural	134 (17.3)	640 (82.7)	
Urban	515 (20.5)	1992 (79.5)	
Educational level			< 0.001
Basic	417 (27.4)	1107 (72.6)	
Medium	134 (17.0)	654 (83.0)	
Higher	98 (10.2)	867 (89.8)	
Body mass index			< 0.001
Normal weight	187 (15.0)	1061 (85.0)	
Low weight	2 (8.0)	23 (92.0)	
Overweight	259 (19.7)	1053 (80.3)	
Obese	155 (29.3)	374 (70.7)	
Smoking habit			< 0.001
Non-smoker	375 (23.6)	1212 (76.4)	
Former smoker	161 (15.8)	860 (84.2)	
Smoker	106 (17.2)	511 (82.8)	
Occasional smoker	7 (12.5)	49 (87.5)	
Alcohol (daily intake)			0.001
0 SDU	542 (21.0)	2042 (79.0)	
1 SDU	73 (18.3)	326 (81.7)	
2–3 SDUs	26 (10.4)	225 (89.6)	
≥ 4 SDUs	8 (17.0)	39 (83.0)	

*SDU* standard drink unit

*Chi-squared test for each independent variable

In the multivariate analysis, the frequency of NEA-OA phenotype increased with age (being 3.6 times more likely in patients aged 80 s or older versus those between 40 and 49 years) and BMI (Table 3). It was significantly more frequent in women than in men, as well as in the center of Spain, versus the north or the Mediterranean area. NEA-OA phenotype was less frequent in those with a higher level of education. There was no association between NEA-OA phenotype and smoking, or with a rural or urban environment. A lower frequency of NEA-OA phenotype was observed in subjects consuming two or three standard drink units (SDU) of alcohol per day.

**Table 3:** Multivariate analysis of NEA-OA vs subjects without axial OA ≥ 40 years old

	OR (95% CI)	*p* value
Age range (years)		
40–49		
50–59	2.156 (1.614; 2.880)	< 0.001
60–69	3.008 (2.232; 4.054)	< 0.001
70–79	2.809 (2.007; 3.931)	< 0.001
≥ 80	3.646 (2.465; 5.391)	< 0.001
Sex		
Women	2.025 (1.600; 2.563)	< 0.001
Region of Spain		
North		
Mediterranean and Canary Islands	0.944 (0.743; 1.199)	0.637
Center	1.355 (1.059; 1.732)	0.015
Type of municipality		
Urban	1.202 (0.952; 1.519)	0.123
Educational level		
Basic		
Medium	0.740 (0.582; 0.940)	0.014
Higher	0.461 (0.354; 0.599)	< 0.001
Body mass index		
Normal weight		
Low weight	0.427 (0.097; 1.891)	0.263
Overweight	1.346 (1.080; 1.677)	0.008
Obese	2.129 (1.639; 2.765)	< 0.001
Smoking habit		
Non-smoker		
Former smoker	0.839 (0.665; 1.059)	0.139
Smoker	1.102 (0.839; 1.447)	0.484
Occasional smoker	0.836 (0.363; 1.923)	0.673
Alcohol (daily intake)		
0 SDU		
1 SDU	0.904 (0.667; 1.225)	0.516
2–3 SDUs	0.563 (0.360; 0.881	0.012
≥ 4 SDUs	0.931 (0.415;2.092)	0.863

*SDU* standard drink unit

### Axial OA without a peripheral OA (EA-OA phenotype)

Cases with EA-OA phenotype (without peripheral OA) showed similar characteristics to NEA-OA phenotype (Table 1). Most were women (74%) and lived in urban areas (79.6%). The majority had a basic educational level, were overweight and did not have toxic habits.

When comparing EA-OA phenotype with subjects aged 40 or over without OA, we observed that axial OA increased with age (although less markedly between 60 and 79 years) (Table 4). In addition, it was more frequent in women than men, and in the center of Spain than in the north and Mediterranean region. Frequency proved similar in rural and urban municipalities. As the level of education increased, EA-OA phenotype decreased. It was more frequent in obese and overweight patients than in normal weight subjects, with a significance level of 0.062 in the univariate analysis. The frequency of EA-OA phenotype was lower in former smokers and occasional smokers, as well as in subjects who consumed 2–3 SDUs per day.

**Table 4:** Univariate analysis of EA-OA vs subjects without OA ≥ 40 years old

	EA-OA	Subjects without axial OA	*p* value*
Age range (years)			< 0.001
40–49	69 (7.0)	912 (93.0)	
50–59	110 (15.9)	580 (84.1)	
60–69	73 (14.8)	421 (85.2)	
70–79	40 (13.5)	256 (86.5)	
≥ 80	27 (16.1)	141 (83.9)	
Sex			< 0.001
Men	83 (8.1)	945 (91.9)	
Women	236 (14.7)	1365 (85.3)	
Region of Spain			< 0.001
North	85 (11.2)	676 (88.8)	
Mediterranean and Canary Islands	104 (9.6)	979 (90.4)	
Center	130 (16.6)	655 (83.4)	
Type of municipality			0.124
Rural	65 (10.4)	561 (89.6)	
Urban	254 (12.7)	1749 (87.3)	
Educational level			< 0.001
Basic	175 (16.0)	922 (84.0)	
Medium	81 (12.2)	581 (87.8)	
Higher	63 (7.3)	805 (92.7)	
Body mass index			0.062
Normal weight	115 (10.5)	985 (89.5)	
Low weight	2 (8.7)	21 (91.3)	
Overweight	137 (13.1)	910 (86.9)	
Obese	54 (15.3)	299 (84.7)	
Smoking			0.048
Non-smoker	164 (13.7)	1036 (86.3)	
Former smoker	82 (9.8)	759 (90.2)	
Smoker	69 (12.8)	472 (87.2)	
Occasional smoker	4 (8.5)	43 (91.5)	
Alcohol (daily intake)			0.001
0 SDU	270 (13.1)	1795 (86.9)	
1 SDU	37 (11.8)	277 (88.2)	
2–3 SDUs	8 (3.8)	202 (96.2)	
≥ 4 SDUs	4 (10.0)	36 (90.0)	

*SDU* standard drink unit

*Chi-squared test for each independent variable

In the multivariate analysis (Table 5), there was a significant relationship between age and EA-OA phenotype; in the 70–79 age group the increase was slightly lower than in the 50–59 age group, with a peak in those over 80 years. EA-OA phenotype was more frequent in women, in central Spain, in people with low educational levels, as well as in obese subjects. In contrast, it was less frequent in people consuming 2–3 SDUs per day. No relationship between smoking habit and EA-OA phenotype was found.

**Table 5:** Multivariate analysis of EA-OA vs subjects without OA ≥40 years old

	OR (95% CI)	*p* value
Age range (years)		
40–49		
50–59	2.128 (1.526; 2.966)	< 0.001
60–69	1.940 (1.337;2.817)	< 0.001
70–79	1.746 (1.109; 2.749)	0.016
≥ 80	2.430 (1.410;4.186)	0.001
Sex		
Women	1.706 (1.267; 2.295)	< 0.001
Region of Spain		
North		
Mediterranean and Canary Islands	0.824 (0.600; 1.131)	0.231
Center	1.451 (1.062; 1.983)	0.019
Type of municipality		
Urban	1.293 (0.950; 1.759)	0.102
Educational level		
Basic		
Medium	0.807 (0.594; 1.096)	0.170
Higher	0.494 (0.355; 0.687)	< 0.001
Body mass index		
Normal weight		
Low weight	0.676 (0.153; 2.995)	0.606
Overweight	1.308 (0.991; 1.727)	0.058
Obese	1.618 (1.121; 2.334)	0.010
Smoking habit		
Non-smoker		
Former smoker	0.783 (0.578; 1.061)	0.115
Smoker	1.126 (0.808; 1.568)	0.484
Occasional smoker	0.794 (0.274; 2.299)	0.671
Alcohol (daily intake)		
0 SDU		
1 SDU	1.049 (0.710; 1.549)	0.810
2–3 SDUs	0.305 (0.145; 0.641)	0.002
≥ 4 SDUs	0.878 (0.269; 2.302)	0.662

*SDU* standard drink unit

### Cervical OA vs lumbar OA

The prevalence of cervical OA based on clinical or clinical-radiographic criteria was 10.10% (95% CI 9.07–11.24). By sex, it measured 13.90% (12.42–15.54) in women and 5.94% (4.62–7.60) in men. Of the 345 patients with cervical OA, 78.3% had already been diagnosed prior to EPISER2016. Only in the remaining percentage were the new criteria used (Fig. 2). The 118 cases of cervical OA based on clinical-radiographical criteria and without lumbar osteoarthritis were mostly women (73.7%) and lived in urban areas (80.5%). Most had a basic educational level (56.8%), were overweight or obese (55.4%) and did not refer toxic habits (73.7% were non-smokers or ex-smokers and 84.7% did not consume alcohol on a daily basis) (Table 1).

The prevalence of lumbar OA based on clinical or clinical-radiographic criteria was 15.52% (95% CI 14.30–16.83). By sex, it was 21.03% (19.29–22.88) in women and 9.48% (7.85–11.40) in men. Of the 540 patients with lumbar OA, 80.6% had already been diagnosed prior to EPISER2016; thus, the new diagnostic criteria were applied in 19.4% of cases (105/540) (Fig. 2). The 310 subjects with lumbar OA based on clinical-radiographic criteria and without cervical osteoarthritis presented characteristics similar to those cases with cervical osteoarthritis and without lumbar osteoarthritis: most were women (75.2%), lived in an urban environment (79.7%), had a basic educational level, were overweight or obese and did not refer toxic habits (Table 1).

The univariate analysis between patients with cervical OA and patients with lumbar OA showed (Table 6) that the difference between cervical and lumbar OA (there was a higher frequency of the latter) increased with age, with BMI, in North and Central Spain versus the Mediterranean region, and in patients who did not smoke or who were ex-smokers; these increases were statistically significant. The difference between the frequency of lumbar and cervical OA was similar in men and women and was not influenced by the type of municipality (rural or urban). It was somewhat lower in those with a higher educational level, but this decrease was not statistically significant.

**Table 6:** Univariate analysis of cervical OA vs lumbar OA

	Cervical OA	Lumbar OA	*p* value*
Age range (years)			0.012
40–49	25 (35.2)	46 (64.8)	
50–59	41 (33.3)	82 (66.7)	
60–69	31 (29.8)	73 (70.2)	
70–79	12 (16.0)	63 (84.0)	
≥ 80	9 (16.4)	46 (83.6)	
Sex			0.760
Men	31 (28.7)	77 (71.3)	
Women	87 (27.2)	233 (72.8)	
Region of Spain			0.013
North	27 (22)	96 (78)	
Mediterranean and Canary Islands	54 (36.2)	95 (63.8)	
Center	37 (23.7)	119 (76.3)	
Type of municipality			0.848
Rural	23 (26.7)	63 (73.3)	
Urban	95 (27.8)	247 (72.2)	
Educational level			0.177
Basic	67 (26.3)	188 (73.7)	
Medium	23 (24.2)	72 (75.8)	
Higher	28 (35.9)	50 (64.1)	
Body mass index			0.005
Normal weight	48 (35.6)	87 (64.4)	
Low weight	2 (100)	0	
Overweight	44 (25.9)	126 (74.1)	
Obese	18 (19.4)	75 (80.6)	
Smoking habit			0.018
Non-smoker	58 (24.7)	177 (75.3)	
Former smoker	29 (26.1)	82 (73.9)	
Smoker	31 (40.8)	45 (59.2)	
Occasional smoker	0	6 (100)	
Alcohol (daily intake)			0.966
0 SDU	100 (27.8)	260 (72.2)	
1 SDU	12 (26.7)	33 (73.3)	
2–3 SDUs	4 (23.5)	13 (76.5)	
≥ 4 SDUs	2 (33.3)	4 (66.7)	

*SDU* standard drink unit

*Chi-squared test for each independent variable

In the multivariate analysis (Table 7), the difference between the frequency of lumbar OA and cervical OA was greater with increasing age (this increase was statistically significant in the older groups) and was not associated with gender. In the Mediterranean region and the Canary Islands, the difference between the frequency of lumbar and cervical OA was less than in the north and center of Spain; this decrease was statistically significant. In overweight and obese subjects, the difference between the frequency of lumbar OA and cervical OA was greater than in those of normal weight, being statistically significant for obesity.

**Table 7:** Multivariate analysis of cervical OA vs lumbar OA

	OR (95% CI)	*p* value
Age range (years)		
40–49		
50–59	1.033 (0.543; 1.965)	0.920
60–69	1.207 (0.606; 2.404)	0.592
70–79	2.381 (1.026; 5.523)	0.043
≥ 80	2.446 (0.963; 6.212)	0.060
Sex		
Women	1.234 (0.731; 2.081)	0.431
Region of Spain		
North		
Mediterranean and Canary Islands	0.45 (0.253; 0.800)	0.007
Center	0.921 (0.509; 1.667)	0.785
Body mass index		
Normal weight		
Overweight	1.638 (0.974; 2.756)	0.063
Obese	2.066 (1.076; 3.967)	0.029

## Discussion

In this paper, we analyzed the main characteristics of axial OA based on EPISER2016, a cross-sectional multicenter population-based study. Our data showed that the prevalence of axial OA based on clinical or clinical-radiographic criteria in subjects aged 40 or more years was 19.17% (95% CI: 17.82–20.59). To our knowledge, this is the first data on axial OA (cervical and/or lumbar OA) in Spain and, as far as we know, the first study on the prevalence of axial OA in Europe.

In this project, we compared two populations of patients with axial OA vs subjects aged 40 years or more without axial OA. The first group was comprised of subjects with axial OA based on clinical-radiographic criteria who might have peripheral OA (NEA-AO phenotype, a total of 649 cases). The second group consisted of exclusive axial OA (EA-AO); these were subjects with axial OA based on clinical-radiographic criteria but without peripheral OA (319 cases).

In the multivariate analysis of patients with NEA-OA phenotype versus subjects in the same age range without axial OA, OA increased with age and was more frequent in women. The relation between sex and axial OA is not clear in previous papers. In some studies, higher prevalence was associated with male sex [12], in others it was linked to female sex [13, 14], while in still others there was no significant difference [7, 15, 19]. This probably reflects the influence of different factors (ex. Genetics and racial characteristics). NEA-OA phenotypes was more prevalent in people with a lower level of education, which could reflect the greater likelihood of physical labor-like work [27]. The prevalence was higher in the center of Spain than in the north or Mediterranean area. Although peripheral OA has been linked to geographic areas (e.g., knee OA in Africa or hip OA in Asia), no association has been described between axial OA and this factor. A lower frequency of NEA-OA phenotype was observed in subjects who consumed 2 to 3 units of alcohol per day. To our knowledge, this is the first description of axial OA and alcohol consumption.

The same results were observed when in the multivariate analysis comparing subjects with EA-OA phenotype versus those without axial OA. The only difference between the two Axial OA phenotypes is that the prevalence of NEA-OA increased significantly in those who were overweight or obesity, while EA-OA only increased significantly with obesity. These results are interesting because they suggest that biomechanical factors are relevant in axial OA. Lumbar OA was associated with obesity in other studies [12, 13]; e.g., Hasset et al. described an increase of lumbar OA in association with BMI, but with borderline significance for obesity [28].

Lumbar OA was more frequent than cervical OA (prevalence of 15.52%, 95% CI 14.30–16.83, and 10.10%, 95% CI 9.07–11.24, respectively). There are to the best of our knowledge no published data on the prevalence of axial OA including both cervical and lumbar OA, and data on the prevalence of cervical and lumbar OA, separately, are scarce. Both show great variability, from 3.38% to 20.46% in cervical OA [20, 21], and from 5.6 to 67% in lumbar OA [7, 14]. One publication that examined lumbar OA in corpses showed that it was present in 100% of adults over 60 years of age [19]. Crude and adjusted prevalence rates of lumbar OA in subjects older than 18 years in Beijing was 9.02 and 7.44%, respectively [13]. These data could not be directly compared with EPISER2016 because our results refer to a population aged 40 years or older and imaging examinations (radiography, computerized tomography (CT) or magnetic resonance) were mandatory for diagnosing lumbar osteoarthritis in the Chinese study. The prevalence of clinical-radiographic axial OA in Korea was 16% in women and 5.6 in men [14], but any comparison with EPISER2016 is also problematic because the former involved a population aged 50 years or more and the pain duration used to define OA was more than a month (in EPISER2016 it was more than three months). Another Korean study based on computerized tomography in people over 20 years old observed a radiographic lumbar OA prevalence of 20.23% in men and 14.29% in women [15].

When comparing cervical and lumbar OA, the latter was more common. The difference between the two was significantly greater in those aged 70 years or more and in the obese. It was also greater in northern and central Spain than in the Mediterranean area; we have no clear explanation for this result.

As regards the limitations of the study, some factors that could influence the prevalence of axial OA, such as the type of work that people do (prolonged hours in the same position, weight bearing…) were not available for our analysis. The lack of validated criteria for the diagnosis of cervical or lumbar OA was also a limitation. Lumbar OA has been described as typically affecting the anterior structures first and then later the posterior ones. There are, however, are atypical patterns of disease [5]. In the published articles on lumbar OA, it is described in relation to K-L involvement, according to osteophytes and the narrowing of intervertebral disc space [12]. However, there are no validated criteria for the diagnosis. For this reason, EPISER2016 clinical-radiographic diagnostic criteria were defined, to include pain, stiffness and at least one radiological criterion (osteophytes or space reduction, or sclerosis in interapophyseal joints). These criteria were only used in patients with no prior diagnosis (75 subjects with cervical OA out of 345, and 105 out of 540 with lumbar OA). We reanalyzed the data excluding patients diagnosed by these new criteria, and our results did not differ significantly (data not shown).

Another important aspect to consider is the representativeness of the sample. In this regard, the self-reported data on OA chronic cervical pain and chronic lumbar pain available from the 2017 National Health Survey of Spain, which boasts rigorous sampling procedures, are similar to those that were initially self-reported by the subjects in EPISER2016 (20.6 vs 18.4%; 17.4 vs 13.5%; 21.7 vs 18.4%, respectively). This would indicate that the possible reasons for refusing to participate in EPISER2016 were not associated with its primary objective [26, 29].

In conclusion, this is the first study on the prevalence of axial OA phenotypes in Europe describing the associated socio-demographic, anthropometric, and lifestyle variables. Non-Exclusive Axial OA (NEA-OA Phenotype) was more frequent in women, in people with a lower level of education and in those living in the center of Spain (versus the northern or Mediterranean areas). Exclusive axial OA (EA-OA phenotype) increased with obesity. Lumbar OA was more prevalent than cervical OA, this difference being significantly greater in adults aged 70 years or more and in obese subjects.

Characterizing the two axial OA phenotypes is important because it can help us understand the different risk factors associated with them as well as possible differences in the pathogenetic mechanisms of the two phenotypes. All of this can influence treatment. It will also help select patients for possible clinical trials. Peripheral OA and axial OA are clearly two phenotypes of OA. Knowing whether exclusive axial OA (EA-OA) is different from axial OA associated with peripheral OA (NEA-OA) can help us to study its risk factors, pathogenetic mechanisms, prognosis of the disease and also its correct treatment.
